# Identification and Expression of New Unspecific Peroxygenases – Recent Advances, Challenges and Opportunities

**DOI:** 10.3389/fbioe.2021.705630

**Published:** 2021-07-07

**Authors:** Alina Kinner, Katrin Rosenthal, Stephan Lütz

**Affiliations:** Chair for Bioprocess Engineering, Department of Biochemical and Chemical Engineering, TU Dortmund University, Dortmund, Germany

**Keywords:** unspecific peroxygenase (UPO), oxyfunctionalization, fungal enzyme, heme enzyme, genome mining, heterologous expression, high-throughput screening

## Abstract

In 2004, the fungal heme-thiolate enzyme subfamily of unspecific peroxygenases (UPOs) was first described in the basidiomycete *Agrocybe aegerita*. As UPOs naturally catalyze a broad range of oxidative transformations by using hydrogen peroxide as electron acceptor and thus possess a great application potential, they have been extensively studied in recent years. However, despite their versatility to catalyze challenging selective oxyfunctionalizations, the availability of UPOs for potential biotechnological applications is restricted. Particularly limiting are the identification of novel natural biocatalysts, their production, and the description of their properties. It is hence of great interest to further characterize the enzyme subfamily as well as to identify promising new candidates. Therefore, this review provides an overview of the state of the art in identification, expression, and screening approaches of fungal UPOs, challenges associated with current protein production and screening strategies, as well as potential solutions and opportunities.

## Introduction

The selective oxyfunctionalization of organic molecules is one of the most challenging tasks in synthetic chemistry. Biotransformations are used in the pharmaceutical industry to replace complex chemical syntheses, e.g., to obtain drug metabolites for pharmacological activity and toxicity studies (Rosenthal and Lütz, 2018). Especially, the large group of heme enzymes harbors versatile biocatalysts, which oxidize non-activated C-H bonds regio- and stereospecifically under mild reaction conditions (Schmitz et al., 2019a). In 2004, a new type of heme-thiolate enzyme with mono(per)oxygenase activity was discovered in the basidiomycetous fungus *Agrocybe aegerita* catalyzing a broad range of oxidative transformations (Ullrich et al., 2004). These aromatic peroxygenases, later renamed as unspecific peroxygenases (UPOs, E.C. 1.11.2.1), are extracellular fungal enzymes, which form one among five members of a new sub-subclass of oxidoreductases (E.C. 1.11.2.-). Unlike the related group of chloroperoxidases (CPOs, E.C. 1.11.1.10), UPOs have minor natural catalytic activity toward chlorination reactions but otherwise efficiently catalyze a broad range of selective oxyfunctionalization of non-activated C–H- and C=C-bonds as well as C–C-bond cleavage. In contrast to cytochrome P450 monooxygenases (P450, E.C. 1.14.-.-), UPOs do not rely on electron donating flavin- or iron-sulfur-containing redox partners; instead they only require H_2_O_2_, which serves as both electron acceptor and oxygen donor, and the peroxygenase can thus be considered as self-sufficient (Ullrich and Hofrichter, 2005). Resting state UPOs contain the ferric heme with a loosely bound water ligand, which is then replaced by hydrogen peroxide to form the peroxo-complex Compound 0 and subsequently the key intermediate Compound I, an oxo-ferryl cation radical complex. This strong oxidant abstracts a hydrogen atom from the substrate, forming the protonated intermediate Compound II and finally releasing the hydroxylated product after rapid recombination of the short-lived substrate radical. Since UPOs can perform one-electron and two-electron oxidations, they are assumed to be the “missing link” between P450s and CPOs from a catalytic perspective (Hofrichter et al., 2015). Several patents on UPO-sequences and UPO-catalyzed reactions demonstrate their potential for industrial application ranging from hydroxylation of aliphatic hydrocarbons to deacylation of corticoids (e.g., Hofrichter et al., 2005, 2011; Pecyna et al., 2008; Landvik et al., 2013; Vind et al., 2014; Holla et al., 2015; Molina-Espeja et al., 2016b; Fernández-Fueyo et al., 2019).

Although UPOs have several advantageous properties, limiting factors have been identified that need to be overcome to render the biocatalysts suitable for industrial applications. For instance, frequently poor selectivity, limited enzyme activity and stability under process conditions as well as low substrate and co-substrate loadings are main issues to consider and solve (Bormann et al., 2015; Hobisch et al., 2020). In addition, UPO-catalyzed biotransformations require sufficient supply of the oxidant for substrate oxidation. However, the oxidizing agent H_2_O_2_ can inhibit the activity of peroxygenases and irreversibly inactivate the UPO, e.g., by degradation of the prosthetic heme group (Karich et al., 2016). The destructive effect on heme enzymes is typically observed when a larger excess of the oxidant is present (Valderrama et al., 2002). Therefore, the *in situ* generation of H_2_O_2_ by catalytic O_2_ reduction is the most common method balancing efficient peroxygenase activity and H_2_O_2_-induced inactivation. Next to enzymatic or chemical supply of H_2_O_2_, electrochemistry and photocatalysis are promising approaches, which have been developed (Lütz et al., 2004; Churakova et al., 2011; Horst et al., 2016; Ni et al., 2016; Schmitz et al., 2017). Moreover, limited solubility of the substrates due to their hydrophobicity is a challenging issue, which can be addressed, for example, by reaction engineering. In this context, several authors reviewed recent developments as well as challenges and opportunities of peroxygenase-catalyzed reactions regarding protein, reaction, and medium engineering approaches in detail (Bormann et al., 2015; Wang et al., 2017; Hobisch et al., 2020; Aranda et al., 2021). Furthermore, Sigmund and Poelarends provided an overview of the current state of enzymes with peroxygenase activity focusing on engineering strategies to improve oxyfunctionalization reactions (Sigmund and Poelarends, 2020). For a general overview of the fungal peroxygenase superfamily, Hofrichter et al. (2020) summarized the state of knowledge of basic and applied UPO research including phylogeny, protein structure, and catalytic activity.

The aim of this contribution was therefore to review the current state of the art in UPO identification, recombinant expression, and screening approaches along with a brief overview of the status of the sustainable biotechnological applicability of UPOs. In particular, the following tools for selection of putative UPOs as well as UPO synthesis and characterization are discussed: (1) genome mining approaches to identify new candidates, (2) efficient expression systems for protein production, and (3) high-throughput methods for time-saving evaluation of enzyme activity.

## Genome Mining Approaches to Identify New UPOs

Due to their promiscuity for oxygen transfer reactions, UPOs have been focused on both in basic and applied research. According to phylogenetic analysis, UPOs can generally be divided into two families: Family I, which includes “short-type” UPOs, and family II containing “long-type” UPOs (Kellner et al., 2014; Hofrichter et al., 2015). To date, family I comprises more than 1,100 short-type UPOs, occurring in most fungal phyla, with a mean size of around 30 kDa, but mainly without predictable signal peptides. Nonetheless, signal peptide-containing and thus secreted UPOs such as the characterized UPOs from *Chaetomium globosum* (*Cgl*UPO), *Marasmius rotula* (*Mro*UPO) and *Marasmius wettsteinii* (*Mwe*UPO) as well as well-established chloroperoxidase from *Leptoxyphium* (*Caldariomyces*) *fumago* (*Lfu*CPO, also known as *Cfu*CPO) are assigned to this short-type family (Hofrichter et al., 2020). Both characterized *Marasmius*-UPOs are assumed to be dimeric proteins linked by intermolecular disulfide bridges to connect the monomers. Intramolecular bridges, however, are not present (Olmedo et al., 2017; Ullrich et al., 2018). Family II harbors more than 900 long-type UPOs along with the well-studied model enzyme *Aae*UPO from *Agrocybe aegerita.* Family II UPOs have a mean size of 44.4 kDa and can only be found in *Ascomycota* and *Basidiomycota*. Here, most sequences possess a C-terminal disulfide bridge and predicted signal peptides, which indicates the extracellular location of the protein. Both UPO families contain highly conserved amino acid motifs essential for their catalytic functionality. The motifs -EHD-S-E- and -EGD-S-R-E- are found in family I and II, respectively, along with the characteristic PCP motif in both families. On the other hand, there are several structural differences in UPO architecture, such as the heme access channel, which might explain the differences in substrate specificity (Hofrichter et al., 2020). For instance, neither *Mro*UPO nor *Aae*UPO convert the sterically demanding compound testosterone, while the recently identified *Cgl*UPO is able to oxidize the steroid molecule (Kiebist et al., 2017).

Recently, Faiza et al. (2019a) searched for novel UPOs by genome mining of more than 800 fungal genomes and identified 113 putative sequences in 35 fungal species. Based on phylogenetic analysis and motif patterns, a new classification of UPOs with five subfamilies was proposed, in which *Aae*UPO and *Cci*UPO from *Coprinopsis cinerea* belong to subfamily I, while *Lfu*CPO and *Mro*UPO are classified in a separate peroxidase-peroxygenase (Pog) superfamily between classical CPOs and UPOs. Furthermore, they created the first UPO online database called “Unspecific Peroxygenase Database” (UPObase) that provides more than 1,900 putative UPO protein sequences including information about classification and motifs as well as homology search tools such as multiple sequence alignments and phylogenetic trees (Faiza et al., 2019b). However, the complex phylogenetic organization of UPO genes and the physiological background of certain amino acid residues are not yet completely understood.

**FIGURE 1:**
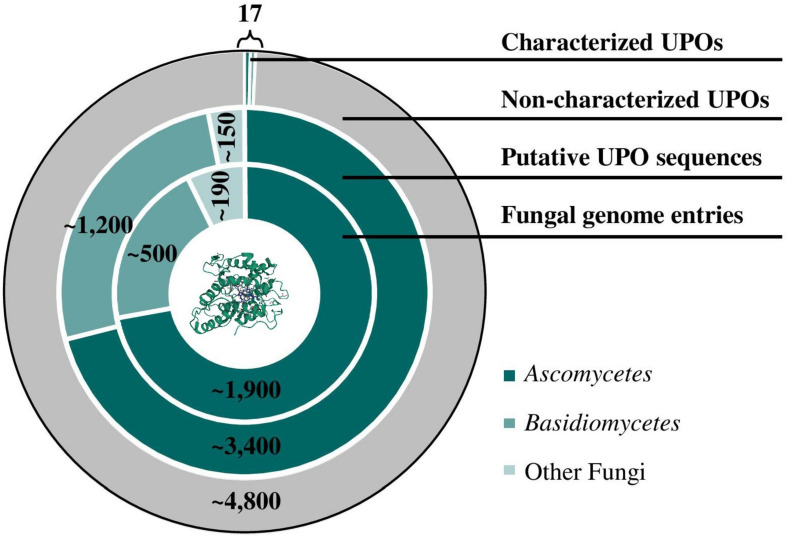
Characterization gap of UPO proteins. Database searches for putative UPO sequences were performed by matching the conversed heme haloperoxidase family profile (“PS51405”) deposited on PROSITE with protein sequences stored in the UniProtKB protein database. Nine ascomycetous and eight basidiomycetous UPOs (excluding protein isoforms) have been purified and characterized to date. Currently, approximately 2,700 fungal genome entries are listed in NCBI. (PDB ID 5OXU; Ramirez-Escudero et al., 2018).

At a molecular level, crystal structures of well-studied model enzymes *Lfu*CPO (1CPO; Sundaramoorthy et al., 1995), *Aae*UPO (2YOR; Piontek et al., 2013), *Mro*UPO (5FUJ; Piontek et al., 2017) as well as laboratory-evolved PaDa-I mutant (5OXU; Ramirez-Escudero et al., 2018) have been elucidated in recent years. Growing knowledge of the protein sequence of UPOs led to the identification of numerous UPO-like genes via database search in sequenced organisms. With an increasing availability of whole genome sequences and thus an increasing number of annotated UPOs, it is no longer a matter of the accessibility of gene sequences but of the correct determination of the gene-function relationship. Figure 1 demonstrates the divergence between the number of genomic sequences annotated as putative UPOs deposited in UniProtKB and the number of verified UPOs over the last two decades. In fact, NCBI currently provides more than 2,500 fungal genomes with thousands of putative UPO sequences mainly in *Ascomycetes* and *Basidiomycetes*. On the contrary, to the best of our knowledge, only nine ascomycetous and eight basidiomycetous UPOs (excluding protein isoforms) have been purified and characterized to date, resulting in more than 4,800 non-characterized peroxygenase sequences. In addition to the wild-type UPO protein, the high number of UPO variants generated by directed evolution approaches (e.g., 9,000 clones by Molina-Espeja et al., 2014) further emphasizes the characterization gap between identified or generated candidates and evaluation of their catalytic activity. Current attempts to close this gap are discussed in the following.

## UPO Production Approaches

Despite the great potential of UPOs for biotechnological purposes, practical application of this novel enzyme subclass harbors several technical bottlenecks, such as the often limited enzyme activity and stability along with frequently very low reactant concentrations (Bormann et al., 2015). In general, a precondition for protein engineering and enzyme application on industrial scale is their homologous or heterologous expression at high yields. Therefore, the application of UPO proteins in the pharmaceutical or fine chemical industry will only be feasible if a cost-effective and reliable biocatalyst production in the “g-per-liter” range is realized. In particular, the widespread yeast workhorse *Pichia pastoris* (syn. *Komagataella phaffii*) produced recombinant mammalian proteins with a titer of up to 20 g L^–1^, while filamentous fungi like *Aspergillus niger* and *Trichoderma reesei* were able to secrete 25 g L^–1^ of glucoamylase (*A. niger*) and 20–30 g L^–1^ of cellulase (*T. reesei*) (Durand et al., 1988; Demain and Vaishnav, 2009). To date, UPOs have been secreted in their native fungal producers (Ullrich et al., 2004, 2018; Anh et al., 2007; Gröbe et al., 2011; Kiebist et al., 2017; Kimani, 2019) or heterologously expressed in *A. niger* (Conesa et al., 2001), *Aspergillus oryzae* (Pecyna et al., 2008), *Escherichia coli* (Carro et al., 2019), *P. pastoris*, and *Saccharomyces cerevisiae* (Molina-Espeja et al., 2014, 2015) at the “mg-per-liter” level. Major challenges for efficient UPO synthesis are certainly the signal peptide (e.g., insufficient interaction between signal recognition particle (SRP) and hydrophobic region of the signal peptide), post-translational modifications (e.g., high degree of glycosylation) and the lack of chaperones in especially heterologous host organisms. Interestingly, natural hyperglycosylation by *S. cerevisiae* appeared to have a beneficial effect on enzyme activity of evolved *Aae*UPO mutants, probably due to increased stability, as well as optimization of the 43-amino-acid signal peptide led to markedly enhanced protein secretion (Molina-Espeja et al., 2014). When expressed in prokaryotic *E. coli*, codon optimization of the fungal gene sequence combined with reduced induction using an auto-induction growth medium and a low incubation temperature (16°C) resulted in correctly folded active UPO proteins (Linde et al., 2020).

Table 1 provides a detailed overview of current UPO expression and purification approaches including native and recombinant producers. In addition to the already characterized UPO proteins, UPO activity has been detected without further purification in the fungal species *Agaricus bisporus*, *Agrocybe alnetorum*, *Agrocybe chaxingu*, *Agrocybe parasitica*, *Auricularia auricula-judae*, and *Mycena galopus* (Hofrichter et al., 2015). *In vitro* UPO expression approaches using cell-free protein synthesis technologies have not yet been reported but harbor high potential for high-throughput enzyme screening (Rolf et al., 2019).

**TABLE 1:** Current UPO expression approaches using native and heterologous producer organisms.

				Culture liquid		Purified enzyme					
					
Enyzme	Expression organism	Culture volume [L]	Cultivation duration	Total protein [mg L^–1^]	Specific activity [U mg^–1^]	Total protein [mg L^–1^]	Specific activity [U mg^–1^]	Recovery yield [%]	Protein yield [%]^*b*^	Purification step (purification factor)	References
**Protein expression in native producers**											
*Aae*UPO	*Agrocybe aegerita*	3.9 (BR)	11 days	119.23^a^	3^e^	0.38^a^	165^e^	18	0.32	IEX (55)	Ullrich et al., 2004
*Cgl*UPO	*Chaetomium globosum*	0.2 (SF)	21 days	∼40	0.6^e^	120^a^	12^e^	8.5	0.42	IEX (20)	Kiebist et al., 2017
*Cra*UPO	*Coprinellus radians*	0.2 (total 4 L f.p.)	13 days	188.10^a^	0.83^e^	0.14^a^	38.5^e^	3.3	0.07	SEC (46.2)	Anh et al., 2007
*Cve*UPO	*Coprinus verticillatus*	0.04 (SF)	21 days	n.a.	n.a.	n.p.	n.p.	n.p.	n.p.	n.p.	Anh et al., 2007 (later purified according to Hofrichter et al., 2020)
*Lfu*CPO	*Leptoxyphium fumago*	0.4 (SF)	10–12 days	n.a.	1001.6^f^	n.a.	2,900.10^f^	70.4	24.27	IEX (2.9)	Yazbik and Ansorge-Schumacher, 2010
	*Leptoxyphium fumago*	1.02 (BR)	13 days	∼1,206^g^	n.a.	n.p.	n.p.	n.p.	n.p.	n.p.	Buchhaupt et al., 2011
	*L. fumago* Cf-CPO4	1.02 (BR)	13 days	∼1,950	n.a.	n.p.	n.p.	n.p.	n.p.	n.p.	Buchhaupt et al., 2011
	*Marasmius rotula*	0.2 (SF)	28 days	445	n.a.	n.p.	n.p.	n.p.	n.p.	n.p.	Gröbe et al., 2011
	*Marasmius rotula*	4 (0.468 L f.p.)	24 days	280	16^e^	2.09^a^	76^e^	0.7	0.13	IEX (4.8)	Gröbe et al., 2011
*Mro*UPO											
*Mwe*UPO	*Marasmius wettsteinii*	0.2 (SF)	21 days	11,320^a,c^	1.6^e^	120^a^	37.5^e^	26	1.06	IEX (24)	Ullrich et al., 2018
*Pab*UPO	*Psathyrella aberdarensis*	15 (BR)	17 days	77.40^a^	2.4^e^	0.20^a^	73.7^e^	7	0.26	IEX (30.8)	Kimani, 2019
**Heterologous protein expression**											
r*Aae*UPO	*Saccharomyces cerevisiae*	1	72 h	∼0.007	n.a.	n.p.	n.p.	n.p.	n.p.	n.p.	Molina-Espeja et al., 2014
	*Pichia pastoris*	n.a.	n.a.	n.d.	n.a.	n.a.	n.a.	n.a.	n.a.	n.a.	Molina-Espeja et al., 2015
r*Cgl*UPO	*Saccharomyces cerevisiae*	0.5 (SF; total 1 L f.p.)	72 h	n.a.	n.a.	0.6	n.a.	n.a.	n.a.	AC	Püllmann et al., 2021
	*Pichia pastoris*	0.5 (SF; total 1 L f.p.)	72 h	n.a.	n.a.	9	n.a.	n.a.	n.a.	AC	Püllmann et al., 2021
r*Gm*aUPO	*Saccharomyces cerevisiae*	0.5 (SF; total 1 L f.p.)	72 h	n.a.	n.a.	n.a.	n.a.	n.a.	n.a.	n.a.	Püllmann et al., 2021
r*Mfe*UPO	*Pichia pastoris*	0.5 (SF; total 1 L f.p.)	72 h	n.a.	n.a.	6.5	n.a.	n.a.	n.a.	AC	Püllmann and Weissenborn, 2021
r*Mhi*UPO	*Pichia pastoris*	0.5 (SF; total 1 L f.p.)	72 h	n.a.	n.a.	5.7	n.a.	n.a.	n.a.	AC	Püllmann and Weissenborn, 2021
r*Mth*UPO	*Saccharomyces cerevisiae*	0.5 (SF; total 1 L f.p.)	72 h	n.a.	n.a.	5	n.a.	n.a.	n.a.	AC	Püllmann et al., 2021
	*Pichia pastoris*	0.5 (SF; total 1 L f.p.)	72 h	n.a.	n.a.	24	n.a.	n.a.	n.a.	AC	Püllmann et al., 2021
r*Mwe*UPO	*Saccharomyces cerevisiae*	0.5 (SF; total 1 L f.p.)	72 h	n.a.	n.a.	n.a.	n.a.	n.a.	n.a.	n.a.	Püllmann et al., 2021
PaDa-I (r*Aae*UPO variant)	*Saccharomyces cerevisiae*	1	72 h	0.14	25^d^	0.21	828^d^	n.a.	n.a.	IEX (33.1)	Molina-Espeja et al., 2014
PaDa-I (r*Aae*UPO variant)	*Saccharomyces cerevisiae*	0.275 (BR; fed-batch)	6 days	n.a.	n.a.	n.a.	n.a.	n.a.	n.a.	n.a.	Molina-Espeja et al., 2015
	*Pichia pastoris*	∼4.4 (BR; fed-batch)	6 days	217	n.a.	∼8	n.a.	n.a.	n.a.	n.a.	Molina-Espeja et al., 2015
	*Pichia pastoris*	0.5 (SF; total 1 L f.p.)	72 h	n.a.	n.a.	12.6	n.a.	n.a.	n.a.	AC	Püllmann and Weissenborn, 2021
r*Cci*UPO	*Aspergillus oryzae*	n.a.	n.a.	n.a.	n.a.	n.a.	100^e^	n.a.	n.a.	n.a.	Babot et al., 2013
r*Cvi*UPO	*Escherichia coli*	3	4–5 days	n.a.	n.a.	n.a.	38.2^d^	25	n.a.	n.a.	González-Benjumea et al., 2020
	*Escherichia coli*	8	4–5 days	831.25^a^	0.17^d^, 0.01^e^	7	38.18^d^, 1.7^e^	27	0.12	Ultrafiltration (25)	Linde et al., 2020
r*Dca*UPO	*Escherichia coli*	10	4–5 days	423^a^	0.06^d^, 0.01^e^	2.8	7.68^d^, 1.62^e^	13	0.09	Ultrafiltration (103)	Linde et al., 2020
	*Pichia pastoris*	0.5 (SF; total 1 L f.p.)	72 h	n.a.	n.a.	16.3	n.a.	n.a.	n.a.	HIC	Püllmann and Weissenborn, 2021
r*Hin*UPO	*Aspergillus oryzae*	n.a.	n.a.	n.a.	n.a.	n.a.	5.4^e^	n.a.	n.a.	n.a.	Peter et al., 2014
r*Lfu*CPO	*Aspergillus niger*	0.5 (SF; total 1 L f.p.)	48 h	10	n.a.	n.a.	39,000 U L^–1^ ^f^	11	n.a.	GPC	Conesa et al., 2001
r*Mro*UPO	*Escherichia coli*	n.a.	4 days	n.a.	n.a.	n.a.	n.a.	n.a.	n.a.	n.a.	Carro et al., 2019
	*Saccharomyces cerevisiae*	0.5 (SF; total 1 L f.p.)	72 h	n.a.	n.a.	0.35	n.a.	n.a.	n.a.	AC	Püllmann et al., 2021
	*Pichia pastoris*	0.5 (SF; total 1 L f.p.)	72 h	n.a.	n.a.	1.1	n.a.	n.a.	n.a.	AC	Püllmann et al., 2021
r*Tte*UPO	*Saccharomyces cerevisiae*	0.5 (SF; total 1 L f.p.)	72 h	n.a.	n.a.	17	n.a.	n.a.	n.a.	AC	Püllmann et al., 2021
	*Pichia pastoris*	0.5 (SF; total 1 L f.p.)	72 h	n.a.	n.a.	21.9	n.a.	n.a.	n.a.	AC	Püllmann and Weissenborn, 2021

*^*a*^Calculated from culture volume.*

*^*b*^Calculated from amount of total protein of culture liquid and purified enzyme.*

*^*c*^Ultrafiltrate.*

*^*d*^ABTS assay.*

*^*e*^Veratryl alcohol assay.*

*^*f*^Monochlorodimedone assay.*

*^*g*^Calculated according to Buchhaupt et al., 2011. *

*AC, affinity chromatography; BR, bioreactor; f.p., for purification; GPC, gel permeation chromatography; HIC, hydrophobic interaction chromatography; IEX, ion exchange chromatography; n.a., not available; n.d., not detectable; n.p., not purified; SEC, size exclusion chromatography; SF, shake flask.*

### Using the Natural Host

Currently, eight enzymes were produced by their respective natural host (see Table 1). Fungal UPO production is routinely conducted in carbon- and nitrogen-rich plant-based media in agitated flasks or stirred-tank bioreactors holding a vessel volume from 100 mL to 5 L (Ullrich et al., 2004; Anh et al., 2007; Gröbe et al., 2011). Therefore, the production of *Pab*UPO from basidiomycetous fungus *Psathyrella aberdarensis* in 30 L scale appears to be an outlier upward among expression approaches using native producers (Kimani, 2019). As frequently observed, medium composition has a crucial influence on the UPO production success (Hofrichter et al., 2015). For instance, UPO-model fungus *A. aegerita* TM A1 significantly varied in UPO expression level when amounts of soybean meal and Bacto Peptone were changed or even when the same fermentation conditions were repeated (Ullrich et al., 2004, 2009). Therefore, for each fungal strain used for UPO production, growth medium and cultivation conditions must be optimized in advance.

Laborious genetic engineering, like construction and transformation of recombinant DNA, is not necessary for the production of unmodified, wild-type UPO proteins with their respective wild-type fungus. Unfortunately, further protein optimization is not possible due to lacking genetic engineering tools for these species. In general, however, the production process is time-consuming because UPO secretion only starts during secondary metabolism phase and reaches its maximum after 2–4 weeks of cultivation. Probably due to non-homogenous growth of most filamentous fungi, biomass was not quantified in the majority of fermentation approaches. Alternatively, maximum enzyme activity serves as an indicator for cultivation termination and harvest, which occurred between 10 and 24 days of fermentation (see Table 1).

As UPOs are extracellular enzymes, protein quantification and activity analysis are possible using the culture supernatant without further cell disruption. Nevertheless, detailed enzyme characterization requires UPO protein purification, which is typically performed in several steps comprising ultrafiltration, multistep fast protein liquid chromatography (FPLC) using anion and cation exchangers as well as size exclusion columns, based on the published method of Hofrichter and colleagues in 2004 (Ullrich et al., 2004). In general, UPO production and subsequent purification appear to be an issue, as the recovery yield of purified enzymes averages 10% combined with low amounts of total protein (∼1% protein yield). So far, the highest yield was achieved with *Mwe*UPO expressed by the saprotrophic basidiomycete *M. wettsteinii* resulting in 26% recovery yield after four FPLC steps (Ullrich et al., 2018). In comparison, five steps were required for purification of *Mro*UPO from related fungus *M. rotula* with an about 40-times lower final yield of 0.7% (Gröbe et al., 2011). However, *Lfu*CPO from fast-growing ascomycete *L. fumago* seems to be an outlier among the other UPO proteins with a recovery yield of 70.4% and a protein yield of ∼24%. Unlike most UPOs, *Lfu*CPO production is routinely conducted in defined fructose-salt medium, while purification is based on aqueous biphasic extraction followed by dialysis and anion exchange chromatography (Pickard, 1981; Yazbik and Ansorge-Schumacher, 2010). In fact, medium and purification optimization were focused to reduce contamination by black pigmentation accompanying cultivation. Efficient and fast purification methods led to the remarkable enzyme recovery with high activity of the chloroperoxidase (purified *Lfu*CPO 2,900 U mg^–1^ toward halogenation of monochlorodimedone). In a different approach, elimination of black pigmentation was addressed by mutagenesis using UV irradiation and resulted in *L. fumago* mutant strain white2 with up to 2.3-fold higher specific productivity and earlier CPO secretion (Buchhaupt et al., 2012). However, to our knowledge, no comparable experiments with other UPO enzymes have been published yet.

In contrast to the widely used model organisms *E. coli*, *S. cerevisiae* and *P. pastoris*, genetic accessibility of fungal UPO-secreting strains is low and only a few molecular tools for *A. aegerita* and *C. cinerea* are available up to now (Sugano et al., 2017; Herzog et al., 2019). In this context, *Lfu*CPO is the only UPO protein expressed by homologous over-expression in the recombinant *L. fumago* strain Cf-CPO4. A CPO-expression cassette was integrated into non-transcribed spacer regions of the ribosomal DNA in *L. fumago* resulting an increase in protein concentration to ∼1.95 g L^–1^ compared to ∼1.2 g L^–1^ in culture liquid of wild-type *L. fumago* (Buchhaupt et al., 2011). Unfortunately, homologous over-expression of *Lfu*CPO was not subject of further published work at this stage. In general, limited or no data on homologous over-expression of UPOs have been available. Therefore, heterologous expression, which allows the biosynthesis of an enzyme from an organism other than its natural producer, has been targeted in several approaches over the last decade.

### Using Heterologous Expression

Since genetical modification of filamentous fungi involves a number of challenges and different transformation techniques are required for different fungal species (Li et al., 2017; Lichius et al., 2020), the current focus is on heterologous protein expression in model organisms to bring versatile UPOs closer to large-scale use and industrial application. First successful attempts of heterologous UPO expression were published almost a decade after the first UPO characterization in 2013 using the mold *A. oryzae* followed by the yeast organism *S. cerevisiae* in 2014 (Babot et al., 2013; Molina-Espeja et al., 2014). Very recently, soluble UPO expression in prokaryotic *E. coli* cells as active enzyme has been reported and further disclosed by an international patent (Fernández-Fueyo et al., 2019), since former efforts of UPO over-expression as bacterial inclusion bodies followed by *in vitro* activation were unsuccessful (Carro et al., 2019; Linde et al., 2020). Excluding the previously conducted genetic engineering work, duration of protein production by heterologous expression could be reduced to 72 h of cultivation using yeast as well as 4–5 days using *E. coli* (see Table 1). In addition, the purification of UPO enzymes can be simplified by using tagged proteins and affinity chromatography. However, recombinant production of wild-type UPOs, without further optimization of the signal peptide, usually varies depending on the protein. For instance, r*Aae*UPO expressed in yeast resulted in almost undetectable amounts of protein (Molina-Espeja et al., 2014, 2015), while r*LfuC*PO was produced in *A. niger* with a titer of ∼10 mg L^–1^ and shake flask production of r*Cgl*UPO and r*Mro*UPO in *P. pastoris* yielded 9 and 1.1 mg L^–1^ enzyme, respectively (Conesa et al., 2001; Püllmann et al., 2021).

Generally, *E. coli* and *S. cerevisiae* are the most popular host organisms for directed evolution mutagenesis methods due to their versatile molecular tools available and their advantageous basis for genetic manipulation (Pourmir and Johannes, 2012). Site-directed mutagenesis was effectively used to alter and improve catalytic activity toward substrates like fatty acids by mutation of neighboring residues at the heme channel in recent approaches. Here, *E. coli* served as expression host for evolved r*Mro*UPO and r*Cvi*UPO variants (Carro et al., 2019; González-Benjumea et al., 2020; Linde et al., 2020). In addition, two wild-type UPOs from the ascomycetes *Collariella virescens* (syn., *Chaetomium virescens*) and *Daldinia caldariorum* were successfully expressed in *E. coli* (2.8–7 mg L^–1^ in culture), purified, and catalytically characterized (Linde et al., 2020). Furthermore, for characterization and modulation of the topography of the active site in UPO enzymes, site-directed mutagenesis and subsequent expression in *S. cerevisiae* revealed the importance of heme channel residues, such as amino acid positions 76, 191 and 241, for substrate accessibility and catalytic activity (Gómez de Santos et al., 2019; Ramirez-Ramirez et al., 2020). In particular, substitutions F191S and G241D in an evolved r*Aae*UPO variant enhanced hydroxylation efficiency toward propranolol (Gómez de Santos et al., 2018), while replacement of F76 by a small residue like alanine had a negative effect on substrate affinity (Ramirez-Ramirez et al., 2020).

Due to inefficient expression capabilities of some UPOs, the optimization of wild-type peroxygenases by directed evolution in yeast has also been pursued in various approaches. Table 2 provides an overview of current mutagenesis attempts in yeast in combination with subsequent high-throughput screening. In general, heterologous expression of UPOs is strongly influenced by their signal peptide, as has been demonstrated during directed evolution studies for improved production of r*Aae*UPO in *S. cerevisiae* by introduction of four advantageous mutations (F[12]Y-A[14]V-R[15]G-A[21]D) that seemed to be beneficial for SRP binding and subsequent processing of the UPO polypeptide (Molina-Espeja et al., 2014). Directed evolution approaches using random as well as recombination-based mutagenesis methods in *S. cerevisiae* led to the three characterized r*Aae*UPO variants PaDa-I (Molina-Espeja et al., 2014), JaWa (based on PaDa-I; Molina-Espeja et al., 2016a), and SoLo (based on JaWa; Gómez de Santos et al., 2018) with SoLo showing improved peroxygenase activity toward propranolol and lower peroxidative activity toward 5′-hydroxypropranolol than the parental enzyme. In a recent approach, Püllmann et al. successfully produced the new wild-type enzymes r*Mfe*UPO from *Myceliophthora fergusii*, r*Mhi*UPO from *Myceliophthora hinnulea*, r*Mth*UPO from *Myceliophthora thermophila* and r*Tte*UPO from *Thielavia terrestris* in *P. pastoris* with production titers between 5.7 and 24 mg L^–1^ after expression optimization using an episomal promoter and signal peptide shuffling system in *S. cerevisiae* (r*Mth*UPO; r*Tte*UPO) or *P. pastoris* (r*Mfe*UPO; r*Mhi*UPO) (Püllmann and Weissenborn, 2021; Püllmann et al., 2021). Currently, the highest titer of recombinant UPO was reported for the PaDa-I variant expressed in *P. pastoris* with 217 mg L^–1^ in fed-batch fermentation, while cultivation of the same *P. pastoris* strain in shake flask yielded only ∼8 mg L^–1^ due to lower cell densities (Molina-Espeja et al., 2015). A successful approach in the “g-per-liter” direction used the ascomycetous mold *A. oryzae* as fungal host to produce r*Cci*UPO from *Coprinopsis cinerea* (Hofrichter et al., 2020). Among others, Novozymes A/S (Denmark) heterologously expresses r*Cci*UPO as well as r*Hin*UPO (rNOVO) from *Humicola insolens* at a scale of 0.2 to 15,000 L, protected by an international patent (Pecyna et al., 2008; Babot et al., 2013; Peter et al., 2014). Growing interest in fungal peroxygenases due to their catalytic potential will likely lead to optimized expression processes toward the “g-per-liter” level, e.g., by strain and reaction engineering, in the future. Consequently, this could also increase the availability of these enzymes, as so far only *Lfu*CPO and evolved *Aae*UPO mutant libraries marketed by EvoEnzyme are commercially available.

**TABLE 2:** High-throughput screening approaches for UPO mutant library analysis.

Directed evolution mutagenesis method	Parental enzyme	Expression host	Screened clones	Assay method	References
Error-prone PCR; *in vivo* DNA shuffling; *in vivo* assembly of mutant libraries; MORPHING; site-directed mutagenesis	r*Aae*UPO	*S. cerevisiae*	∼9,000	ABTS; NBD	Molina-Espeja et al., 2014
Error-prone PCR; *in vivo* DNA shuffling	Evolved r*Aae*UPO PaDa-I	*S. cerevisiae*	∼7,000	ABTS	Martin-Diaz et al., 2018
MORPHING; saturation mutagenesis	Evolved r*Aae*UPO PaDa-I	*S. cerevisiae*	∼4,500	ABTS; NBD	Mate et al., 2017
Error-prone PCR; staggered extension process recombination combined with *in vivo* shuffling	Evolved r*Aae*UPO PaDa-I	*S. cerevisiae*	∼4,000	Naphthalene-Fast Red; DMP	Molina-Espeja et al., 2016a
Combined promoter and signal peptide shuffling system	r*Aae*UPO; r*Dca*UPO; r*Mfe*UPO; r*Mhi*UPO; r*Mth*UPO; r*Tte*UPO	*P. pastoris*	∼3,200	NBD; DMP	Püllmann and Weissenborn, 2021; Püllmann et al., 2021
MORPHING; saturation mutagenesis	Evolved r*Aae*UPO JaWa	*S. cerevisiae*	∼3,000	4-aminoantypirine (4-AAP)	Gómez de Santos et al., 2018
Golden Mutagenesis (Püllmann et al., 2019)	r*Mth*UPO	*S. cerevisiae*	∼900	Octane; cyclohexane; cyclohexene (MISER-GC-MS)	Knorrscheidt et al., 2021
Shuffled peroxygenase gene library	PaDa-I; r*Gma*UPO; r*Cci*UPO	*S. cerevisiae*	672 (screened in 7 h)	1,2,3,4-tetrahydronaphthalene (MISER-GC-MS)	Knorrscheidt et al., 2019, 2020
MORPHING; *in vivo* assembly of mutant libraries; site-directed mutagenesis	r*Aae*UPO	*S. cerevisiae*	∼500 per library	ABTS; NBD	Gonzalez-Perez et al., 2014

*MORPHING, mutagenic organized recombination process by homologous *in vivo* grouping; MISER, multiple injections in a single experimental run.*

## Current UPO Screening Strategies and Analytical Methods

Screening of large enzyme libraries, which consist of protein variants or are derived from natural gene diversity, requires efficient tools applicable in biological matrices. The majority of screening assays rely on changes in absorption or fluorescence during conversion of a model substrate or on indirect sensor systems via coupled enzyme cascade reactions. These assays are mostly substrate or enzyme specific. The substances used, however, often differ from the actual substrate of interest (Kazlauskas, 2006). Therefore, more specific and comprehensive analytics are implemented such as high-performance liquid chromatography (HPLC), gas chromatography (GC) or mass spectrometry (MS) to overcome this limitation. Both approaches entail advantages and disadvantages. Colorimetric or spectrophotometric assays are simple and applicable in every lab. These assays rely on a wavelength shift during the reaction progress, based either on the used model substrate or of indirect measurements of a coupled enzyme cascade reaction or H_2_O_2_ depletion (Johannes et al., 2006; Reymond et al., 2009). In contrast, chromatographic assays are time-consuming and require specialized equipment but allow measurements beyond model substrates (Geueke and Kohler, 2010). Especially spectrophotometric assays were used to date to identify novel UPOs or to describe the reaction kinetics. Tables 3, 4 give an overview about the substrates and products used for colorimetric and spectrophotometric assays. In addition to these reactions, UPOs naturally catalyze a whole range of oxyfunctionalization reactions. More than 400 substrates are now known to be converted by UPOs (Hobisch et al., 2020). These reactions range from aromatic oxidations (Ramirez-Ramirez et al., 2020), epoxidation of aromatics (Zhang et al., 2021), oxidation of alcohols (Kiebist et al., 2017), dealkylation (Püllmann and Weissenborn, 2021), to oxygenation of unsaturated fatty acids (Linde et al., 2020) and halogenations (Ullrich et al., 2004).

**TABLE 3:** Overview of spectrophotometry-based UPO assays.

Substrate	Product	Reaction type	Analyzed enyzme	Specific activity [U mg^–1^]	References
**Spectrophotometry-based assay**					
Veratryl alcohol	Benzaldehyde	Alcohol oxidation	*Aae*UPO	234	Ullrich et al., 2004
				x	Kluge et al., 2007
			*Cgl*UPO	x	Kiebist et al., 2017
			*Cra*UPO	x	Anh et al., 2007
			*Cve*UPO	x	Anh et al., 2007
			*Lfu*CPO	18.1	Ullrich et al., 2004
			*Mro*UPO	x	Gröbe et al., 2011
			PaDa-I (r*Aae*UPO variant)	x	Molina-Espeja et al., 2015; Martin-Diaz et al., 2018
			r*Cvi*UPO	x	Linde et al., 2020
			r*Dca*UPO	x	Linde et al., 2020
Monochloro-dimedone (MCD)	Monochloro-bromodimedone	Bromination	*Aae*UPO	354.3	Ullrich et al., 2004
			*Lfu*CPO	2,859	Ullrich et al., 2004
Monochloro-dimedone (MCD)	Dichloro-dimedone	Chlorination	*Aae*UPO	71.8	Ullrich et al., 2004
			*Lfu*CPO	1,537	Ullrich et al., 2004
Naphthalene	1-Naphtol	Aromatic oxygenation	*Aae*UPO	x	Ullrich and Hofrichter, 2005; Kluge et al., 2007; Ullrich et al., 2009
				217	Kluge et al., 2009
			*Cgl*UPO	x	Kiebist et al., 2017
			*Cra*UPO	x	Anh et al., 2007
			*Cve*UPO	x	Anh et al., 2007
			*Mro*UPO	x	Gröbe et al., 2011
			PaDa-I (r*Aae*UPO variant)	x	Ramirez-Ramirez et al., 2020
			r*Cvi*UPO	x	Linde et al., 2020
			r*Dca*UPO	x	Linde et al., 2020
Veratryl alcohol	Veratraldehyde	Alcohol oxidation	*Aae*UPO	1.2	Reina et al., 2017
				44	Kiebist et al., 2015
				57	Steinbrecht et al., 2020
				62	Aranda et al., 2009
				63	Peter et al., 2013
				63.5	Kiebist et al., 2017
				74.8	Kluge et al., 2007; Kluge et al., 2009
				75	Ullrich et al., 2008
				82	Karich et al., 2018
				87	Vdovenko et al., 2010
				97	Poraj-Kobielska et al., 2013
				98	Karich et al., 2017
				99.6	Ullrich et al., 2018
				103	Ullrich et al., 2009
				106	Peter et al., 2014
				117	Kinne et al., 2009, 2011; Poraj-Kobielska et al., 2011; Carro et al., 2014
				167	Ullrich et al., 2004
				x	Pecyna et al., 2009; Piontek et al., 2010
			*Cgl*UPO	8.2	Steinbrecht et al., 2020
				12	Kiebist et al., 2017
Veratryl alcohol	Veratraldehyde	Alcohol oxidation	*Cra*UPO	23	Steinbrecht et al., 2020
				25.8	Poraj-Kobielska et al., 2011
				29	Poraj-Kobielska et al., 2013
				35	Aranda et al., 2009
				38.5	Anh et al., 2007
			*Cve*UPO	x	Anh et al., 2007
			*Mro*UPO	25	Poraj-Kobielska et al., 2013; Peter et al., 2014
				26	Ullrich et al., 2018
				28	Karich et al., 2017
				48.1	Kiebist et al., 2017
				61	Kiebist et al., 2015
				76	Gröbe et al., 2011
				77	Yarman et al., 2012
				94	Steinbrecht et al., 2020
			*Mwe*UPO	37.5	Ullrich et al., 2018
			*Pab*UPO	117	Kimani, 2019
			PaDa-I (r*Aae*UPO variant)	112	Horst et al., 2016
				x	Molina-Espeja et al., 2015; Carro et al., 2018; Martin-Diaz et al., 2018; Ramirez-Escudero et al., 2018
			r*Cci*UPO	24	Peter et al., 2014
				38.18	Linde et al., 2020
				∼100	Babot et al., 2013, 2015; Aranda et al., 2018
				x	González-Benjumea et al., 2020
			r*Dca*UPO	7.68	Linde et al., 2020
			r*Hin*UPO	5.4	Peter et al., 2014
				15	Poraj-Kobielska et al., 2013

*x, substrate accepted.*

**TABLE 4:** Overview of colorimetry-based UPO assays.

Substrate	Product	Reaction type	Analyzed enyzme	Specific activity [U mg^–1^]	References
**Colorimetry-based assay**					
2,2′-Azinobis-(3-ethylbenzothiazoline-6-sulfonate) (ABTS)	Green ABTS cation radical	One-electron oxidation	*Aae*UPO	295.7	Ullrich et al., 2004
				x	Kluge et al., 2007; Gómez de Santos et al., 2018
			*Cgl*UPO	x	Kiebist et al., 2017
			*Cra*UPO	x	Anh et al., 2007
			*Cve*UPO	x	Anh et al., 2007
			JaWa (r*Aae*UPO variant)	x	Molina-Espeja et al., 2016a; Gómez de Santos et al., 2018
			*Lfu*CPO	1.2	Ullrich et al., 2004
			*Mro*UPO	x	Gröbe et al., 2011
			*Pab*UPO	x	Kimani, 2019
			PaDa-I (r*Aae*UPO variant)	607	Bormann et al., 2020a
				828	Molina-Espeja et al., 2014
				x	Molina-Espeja et al., 2015, 2019, 2016a; Gómez de Santos et al., 2018; Martin-Diaz et al., 2018; Ramirez-Escudero et al., 2018; Ramirez-Ramirez et al., 2020
			PaDa-I-Cys	740	Molina-Espeja et al., 2019
			r*Aae*UPO	x	Mate et al., 2017; Burek et al., 2019
			r*Cvi*UPO	38.2	González-Benjumea et al., 2020
				x	Linde et al., 2020
			r*Dca*UPO	x	Linde et al., 2020
2,6-dimethoxyphenol (DMP)	Colored coerulignone	One-electron oxidation	*Aae*UPO	99.6	Ullrich et al., 2004
				x	Kluge et al., 2007; Püllmann et al., 2021
			*Cgl*UPO	x	Kiebist et al., 2017
			JaWa (r*Aae*UPO variant)	x	Molina-Espeja et al., 2016a
			*Lfu*CPO	1.9	Ullrich et al., 2004
			*Mro*UPO	x	Gröbe et al., 2011
			*Pab*UPO	x	Kimani, 2019
			PaDa-I (r*Aae*UPO variant)	x	Molina-Espeja et al., 2014, 2015, 2016a; Martin-Diaz et al., 2018; Ramirez-Escudero et al., 2018
			r*Cgl*UPO	x	Püllmann et al., 2021
			r*Dca*UPO	x	Püllmann and Weissenborn, 2021
			r*Gma*UPO	x	Püllmann et al., 2021
			r*Mfe*UPO	x	Püllmann and Weissenborn, 2021
			r*Mhi*UPO	x	Püllmann and Weissenborn, 2021
			r*Mro*UPO	x	Püllmann et al., 2021
			r*Mth*UPO	x	Püllmann et al., 2021
			r*Mwe*UPO	x	Püllmann et al., 2021
			r*Tte*UPO	x	Püllmann et al., 2021
Naphthalene + Fast Red reagent [Fast red TR salt hemi (zinc chloride)]	Red azo dye	Aromatic oxygenation	JaWa (r*Aae*UPO variant)	x	Molina-Espeja et al., 2016a
			*Mro*UPO	x	Gröbe et al., 2011
			*Pab*UPO	x	Kimani, 2019
			PaDa-I (r*Aae*UPO variant)	x	Molina-Espeja et al., 2016a; Martin-Diaz et al., 2018
5-nitro-1,3-benzodioxole (NBD)	Yellow (pH 7)/red (pH > 12) 4-nitrocatechol	Demethylenation	*Aae*UPO	x	Poraj-Kobielska et al., 2012
			*Cgl*UPO	x	Kiebist et al., 2017
			*Cra*UPO	x	Poraj-Kobielska et al., 2012
			*Cve*UPO	x	Poraj-Kobielska et al., 2012
			JaWa (r*Aae*UPO variant)	x	Molina-Espeja et al., 2016a
			*Mro*UPO	x	Poraj-Kobielska et al., 2012; Püllmann et al., 2021
			*Pab*UPO	5.2	Kimani, 2019
			PaDa-I (r*Aae*UPO variant)	x	Molina-Espeja et al., 2014, 2015, 2019, 2016a; Gómez de Santos et al., 2018; Martin-Diaz et al., 2018; Ramirez-Escudero et al., 2018; Ramirez-Ramirez et al., 2020
			PaDa-I-Cys	240	Molina-Espeja et al., 2019
			r*Aae*UPO	x	Mate et al., 2017; Püllmann et al., 2021
			r*Cci*UPO	x	Poraj-Kobielska et al., 2012; Püllmann et al., 2021
			r*Cgl*UPO	x	Püllmann et al., 2021
			r*Gma*UPO	x	Püllmann et al., 2021
			r*Mth*UPO	x	Püllmann et al., 2021; Püllmann and Weissenborn, 2021
			r*Tte*UPO	x	Püllmann et al., 2021; Püllmann and Weissenborn, 2021

*x, substrate accepted.*

**FIGURE 2:**
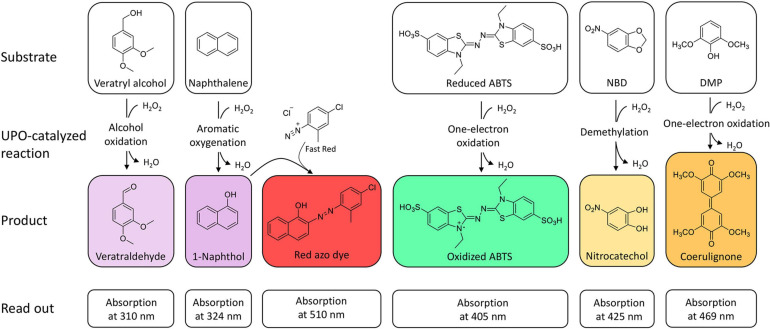
Spectrophotometric assays for the detection and quantification of UPO activity. The products veratraldehyde and 1-naphthol absorb in the non-visible range. Oxidized ABTS, nitrocatechol and coerulignone are visible and can also be used in colorimetric assays.

The oxidation of 2,2′-azino-bis-(3-ethylbenzothiazoline-6-sulfonic acid) (ABTS), 2,6-dimethoxyphenol (DMP), naphthalene, veratryl alcohol, and 5-nitro-1,3-benzodioxole (NBD) are the typical reactions for screening UPOs peroxidase or peroxygenase activity as they are spectrophotometric-detectable reactions (Figure 2). With the substrates ABTS and DMP, the first peroxidase activity has been proven for *A. aegerita* in 2004 (Ullrich et al., 2004). Moreover, DMP was used for further screenings for peroxygenase-like reactions (Molina-Espeja et al., 2016a; Breslmayr et al., 2018). Another assay was reported using NBD as substrate for peroxygenative activity screening. The product 4-nitrocatechol can be quantified at 425 nm over time and turns red after pH shift from 7 to 12, which can be used for end-point determination at 514 nm (Poraj-Kobielska et al., 2012). NBD and ABTS were used in a dual-colorimetric assay to screen five generations of directed evolution comprising more than 9,000 UPO clones (Molina-Espeja et al., 2014). Activity screening with ABTS turned out to be more reliable and stable with a low interference of the culture broth and was therefore used for the first screening round. Interfering background absorption was mainly caused by the presence of hemoglobin. During directed evolution rounds, the screening substrate was changed to NBD, when sufficient amounts of UPOs were secreted. The combination of several screening substrates makes sense in order to circumvent the respective limitations of the used substrate such as sensitivity, specificity or interference with medium compounds. Additionally, the specificity of the substrates leads to application in screenings for either peroxidative activity with ABTS or peroxygenative activity with NBD and the ratio of peroxidative:peroxygenative activity (Mate et al., 2017). A structure-guided evolution of UPOs from PaDa-I led to the modification of the peroxidative:peroxygenative ratio activity indicating the coexistence of various oxidation sites in UPOs. The ability to resolve and modify the peroxygenase and peroxidase activities in a targeted manner will certainly allow to diminish the unwanted peroxidase activity opening a new goal for future protein engineering. Of course, one must always keep in mind that the rules of directed evolution apply, i.e., “you get what you screen for” (Schmidt-Dannert and Arnold, 1999). In case of the optimized variant PaDa-I, it turned out that it also oxides thermodynamically inert compounds such as aromatics providing access to naphthalene epoxides that can be subjected to nucleophilic ring opening reactions yielding chiral trans-disubstituted cyclohexane derivatives (Zhang et al., 2021).

However, background absorption of medium compounds like soybean meal, the key inducing ingredient for UPO production in various fungal species, often leads to detection interferences and thus to limitations in the applicability of spectrophotometric assays (Anh et al., 2007; Poraj-Kobielska et al., 2012). In these cases, supplementation with instrumental analysis is often required. Especially if kinetic parameters are to be determined in addition to screenings (Kluge et al., 2007). Furthermore, substrate and product scope of challenging oxyfunctionalization reactions catalyzed by UPOs can be assessed by using GC or HPLC. The *Aae*UPO turned out to efficiently catalyze the asymmetric benzylic hydroxylation of alkylbenzyl derivatives and C1–C2 epoxidation of styrene derivatives (Kluge et al., 2012). Additionally, the ability to catalyze oxidations at the nitrogen of pyridines is a remarkable property of *Aae*UPO since heterocyclic *N*-oxides are desirable biologically active compounds but efficient and convenient synthetic routes are lacking (Ullrich et al., 2008; Mfuh and Larionov, 2015). Smaller libraries consisting of four UPOs were screened for a two-step conversion of cyclohexane into cyclohexanone (Peter et al., 2014). Cyclohexanone is relevant for polymer synthesis or organic solvent. Especially, the second step from cyclohexanol to cyclohexanone turned out to differ substantially dependent on the used UPO. For larger libraries, instrumental analysis must be adapted to screen sufficient numbers of variants generally in the range of up to several thousands. HPLC- and GC-dependent systems generally require long analysis time. The development of efficient screening methods to identify new UPOs of currently uncharacterized strains and to screen mutant libraries is thus needed. An outstanding approach includes a directed-evolution step coupled to a multiple injections in a single experimental run (MISER)-GC-MS-system (Knorrscheidt et al., 2019, 2020). This system enabled a throughput of a 96-well microtiter plate within 50 min for the analysis of a shuffled library of UPOs heterologously secreted from *S. cerevisiae*. Overall, 672 variants were screened within 7 h on their activity toward 1,2,3,4-tetrahydronaphthalene oxidation resulting the identification of more than 30 novel mutant hits.

While there are a few examples available screening UPO mutant libraries based on heterologous expression systems, there are no high-throughput approaches yet performed to analyze putative UPO-producing wild-type fungi and thus the potential that nature provides us. The natural function of UPOs remains unclear, with different activities proposed, including metabolite synthesis, lignin degradation, and detoxification processes (Hofrichter et al., 2015). Up to now, nine ascomycetous and eight basidiomycetous UPOs were characterized corresponding to 0.4% compared to the uncharacterized putative UPO sequences. In general, compared to high-throughput processes for bacteria and yeast, there has been less progress in developing and validating screening assays for filamentous fungi (Rothschild-Mancinelli et al., 2020). High-throughput screening of not commonly used fungi can have several bottlenecks. These are mainly, amongst others, the non-homogeneous growth in liquid media due to mycelium or pellet formation, low protein production, dependence of protein production on growth condition and medium compounds (Hofrichter et al., 2020; Rothschild-Mancinelli et al., 2020). Nevertheless, screening approaches for fungi were performed for microbial strain libraries containing bacteria and fungi, which were screened on monooxygenase activity using pharmaceutical compounds analyzed with LC-MS (Fredenhagen et al., 2019; Schmitz et al., 2019b). This screening approach is thus available to be transferred to peroxygenase activity screenings in the future, which would be a first step toward medium- to high-throughput screening of natural UPO producing strains. Miniaturization in microwell plate format and automated expression screening has also been successfully performed with filamentous fungi (Alberto et al., 2009). The use of microfluidics and microdroplets can further increase throughput to test millions of biocatalyst candidates. For example, enzyme containing solution or the enzyme producing organism and substrate can be encapsulated in microdroplets and then analyzed with fluorescence-activated sorting methods (Beneyton et al., 2016). Droplet microfluidics combined with electrospray ionization (ESI)-MS can also provide label-free, high-throughput screening with the opportunity to test simultaneously substrate libraries (Diefenbach et al., 2018).

## Conclusion

Fungal UPOs catalyze both, one-electron oxidations (typical peroxidase reactions) and two-electron oxidations with peroxide-derived oxygen transfer (peroxygenation reactions), the latter being more interesting with respect to synthesis of pharmaceuticals. The co-existence of both activities in one biocatalyst, however, leads to lower yields, for instance, due to one-electron oxidation of the desired hydroxylated product. This issue has been successfully addressed by directed evolution in combination with the addition of radical scavengers (Molina-Espeja et al., 2016a; Gómez de Santos et al., 2018). In context of synthesis of phenolic products like 5′-hydroxypropranolol or 4′-hydroxydiclofenac, ascorbic acid has often been used as a radical scavenger since it reduces the formation of unwanted side-products through its oxidation into ascorbyl radical and simultaneous re-reduction of the intermediate phenoxyl radical (Ullrich and Hofrichter, 2007; Poraj-Kobielska et al., 2013; Gómez de Santos et al., 2018). Up to now, only very few approaches have been able to express UPOs at titers in the “g-per-liter” range (Hofrichter et al., 2020). Low stability of UPOs against H_2_O_2_ is typical for all heme-containing enzymes and can be overcome by constant feeding of H_2_O_2_ (Zhang et al., 2017), *in situ* H_2_O_2_ generation (Churakova et al., 2011), or using milder organic peroxides as an alternative (Fernández-Fueyo et al., 2016). Moreover, immobilization strategies have successfully been employed to improve UPO-catalyzed biotransformation through reusability, long-term storage of the enzyme in e.g., cyclohexane, and thus lower cost contribution (Poraj-Kobielska et al., 2015; Fernández-Fueyo et al., 2016; Bormann et al., 2020a). However, UPOs are still in their infancy on the road to large-scale industrial application, and both research and optimization are needed to turn promiscuous enzymes into feasible biocatalysts (Rosenthal and Lütz, 2018; Hobisch et al., 2020; Aranda et al., 2021). In addition to wet lab analysis, computational simulation-based techniques can be used to model and even predict UPO-catalyzed bioprocesses, e.g., in terms of hydrogen peroxide feeding rate (Bormann et al., 2020b; Municoy et al., 2020). Consequently, a combined computational-experimental approach can make enzyme screening more time-saving and efficient.

Regarding the optimization of UPO-catalyzed biotransformations, sustainability and the environmental impact of biochemical reactions are also increasingly coming into focus and gaining importance for process development. Tieves et al. (2019) analyzed the recombinant expression of evolved r*Aae*UPO PaDa-I by *P. pastoris* coupled with the *in situ* H_2_O_2_ generation by the format oxidase *Ao*FOx from *A. oryzae*, expressed by *E. coli*, and evaluated the bienzymatic cascade for hydroxylation of ethyl benzene to (R)-1-phenyl ethanol with respect to the commonly used Sheldon’s E-factor (E for environmental; Sheldon, 2007) as well as the E^+^-factor (classical E-factor plus CO_2_-emissions caused by electricity generation). As a result, their calculation led to an E-factor of 4,300 kg_waste_ kg_product_^–1^ for crude enzyme and 18,500 kg_waste_ kg_product_^–1^ for the purified UPO. Including electricity-related CO_2_ emissions, the E^+^-factors even reached 566,800 kg_waste_ kg_product_^–1^ most likely due to resource-consuming purification steps. As the turnover number (TN) and the protein yield are powerful key factors for the environmental impact of a biocatalyst, increasing the TN of r*Aae*UPO and optimization of the enzyme preparation process can significantly imply lower E-factor contribution (Tieves et al., 2019). Moreover, Bello et al. (2020) recently presented a life cycle assessment (LCA) study of the production and purification of *Cgl*UPO in its native producer *C. globosum* for the enzymatic production of 2,5-furandicarboxylic acid (FDCA) as precursor of bioplastics. Their extensive analysis by impact categories, ranging from land use, human toxicity and ecotoxicity to ozone depletion and global warming, clearly indicated that the main environmental impacts can be attributed to electricity, glucose consumption and culture medium chemicals on laboratory (6 L) and large scale (100,000 L). Among others, polluting or harmful compounds, such as ammonia, nitrates, or methanol, and energy-intensive processes rooted from coal were included in the calculation, revealing critical bottlenecks of the UPO production process (Bello et al., 2020). In summary, studies such as these highlight the importance of identification and characterization of novel UPO enzymes, protein engineering combined with medium- to high-throughput screenings as well as process optimization approaches in both economic and environmental terms.

## Author Contributions

AK, KR, and SL conceptualized and designed the manuscript. AK organized the literature research. AK and KR wrote the manuscript. All authors contributed to manuscript revision, read, and approved the submitted version.

## Conflict of Interest

The authors declare that the research was conducted in the absence of any commercial or financial relationships that could be construed as a potential conflict of interest.
